# Evidence for Cleavage of the Metalloprotease Vsm from *Vibrio splendidus* Strain JZ6 by an M20 Peptidase (PepT-like Protein) at Low Temperature

**DOI:** 10.3389/fmicb.2016.01684

**Published:** 2016-10-25

**Authors:** Rui Liu, Limei Qiu, Qi Cheng, Huan Zhang, Lingling Wang, Linsheng Song

**Affiliations:** ^1^Key Laboratory of Experimental Marine Biology, Institute of Oceanology, Chinese Academy of SciencesQingdao, China; ^2^School of Food Science and Technology, Dalian Polytechnic UniversityDalian, China; ^3^Key Laboratory of Mariculture and Stock Enhancement in North China's Sea, Ministry of Agriculture, Dalian Ocean UniversityDalian, China

**Keywords:** *Vibrio splendidus*, metalloprotease Vsm, peptidase T-like protein, extracellular products, temperature regulation

## Abstract

Metalloprotease Vsm is a major extracellular virulence factor of *Vibrio splendidus*. The toxicity of Vsm from *V*. *splendidus* strain JZ6 has been characterized, and production of this virulence factor proved to be temperature-regulated. The present study provides evidence that two forms (JZE1 and JZE2) of Vsm protein exist in extracellular products (ECPs) of strain JZ6, and a significant conversion of these two forms was detected by SDS-PAGE and immunoblotting analyses of samples obtained from cells grown at 4, 10, 16, 20, 24, and 28°C. Mass spectroscopy confirmed that JZE1 was composed only of the peptidase_M4 domain of Vsm, and JZE2 contained both the PepSY domain and the peptidase_M4 domain. An M20 peptidase T-like protein (PepTL) was screened from the transcriptome data of strain JZ6, which was considered as a crucial molecule to produce the active Vsm (JZE1) by cleavage of the propeptide. Similar to that of Vsm, PepTL mRNA accumulation was highest at 4°C (836.82-fold of that at 28°C), decreased with increasing of temperature and reached its lowest level at 28°C. Deletion of the gene encoding the PepTL resulted in a mutant strain that did not produce the JZE1 cleavage product. The peptidase activity of PepTL recombinant protein (rPepTL) was confirmed by cleaving the Vsm in ECPs with an *in vitro* degradation reaction. These results demonstrate that PepTL participates in activating Vsm in strain JZ6 by proteolytic cleavage at low temperature.

## Introduction

Proteolytic degradation plays important roles in physiological processes of bacteria, including protein maturation, signal peptide modification, gene expression, and nutrients acquisition (Lazdunski, 1989; Charlier et al., 2000). Peptidases have been grouped into 86 families according to their evolutionary relationship, such as serine, cysteine, aspartic, threonine, glutamic-acid and metallo peptidases (Rawlings and Barrett, 1993; Wu and Chen, 2011; Oda, 2012). Among them, the M20 family contains many important members, including metallopeptidases and non-peptidase homologs (amidohydrolases), which are involved in the catalyzed reaction for the release of an N-terminal amino acid from a polypeptide (Rawlings and Barrett, 1995). Members in M20 family commonly exist as homodimers, which contain a zinc-binding domain and a second domain mediating dimerization (Lindner et al., 2003; Chen et al., 2008; Chang et al., 2010). Many M20 peptidases have attracted attention due to their biological functions. For instance, aminopeptidase V (PepV) from *Lactobacillus* sp. and peptidase T (PepT) from *Salmonella typhimurium* are involved in amino acid utilization, and allantoate amidohydrolase from *Escherichia coli* and β-alanine synthase (βAS) from yeast are enzymes of the nucleotides catabolic pathway (Håkansson and Miller, 2002; Jozic et al., 2002; Lundgren et al., 2003; Agarwal et al., 2007).

*Vibrio splendidus* is a pathogen that causes fatal diseases of larval and juvenile marine animals, including turbots (Gatesoupe et al., 1999; Thomson et al., 2005), oysters (Waechter et al., 2002; Garnier et al., 2007), clams (Gómez-León et al., 2005; Kesarcodi-Watson et al., 2009) and scallops (Nicolas et al., 1996; Liu et al., 2013). As an opportunistic pathogen, the virulence of *V*. *splendidus* is mainly regulated by environmental factors, most importantly by temperature (Crapoulet et al., 2006; Wu et al., 2012). *V*. *splendidus* JZ6 is a pathogenic agent of Yesso scallop at the low temperature, and its pathogenicity is significantly reduced when the temperature increases (Liu et al., 2013). The toxicity and the expression of metalloprotease Vsm in extracellular products (ECPs) of *V*. *splendidus* JZ6 are higher at 10°C than that at 28°C (Binesse et al., 2008; Hasegawa et al., 2009; Liu et al., 2016). Furthermore, the toxicity of Vsm was associated with its two protein sizes at different temperatures in ECPs of *V*. *splendidus* JZ6 (Liu et al., 2016), suggesting temperature-dependent post-translational modification of the protease.

Vsm is a multi-domains metalloprotease, containing a signal peptide, a FTP domain, a PepSY domain and two peptidase_M4 domains. The FTP is a fungalysin/thermolysin propeptide domain, which is found in both the bacterial M4 peptidase propeptide and the fungal M36 propeptide to prevent premature activation of proteases (Tang et al., 2003). The PepSY domain is also found in the peptidase M4 family and likely has protease inhibitory activity (Yeats et al., 2004). The peptidase_M4 domain is the metalloprotease activity motif in MEROPS peptidase M4 family with a metal ion binding site (Rawlings and Barrett, 1995). According to its structural feature, the active protein of Vsm must be cleaved to produce the mature enzyme by other peptidase.

In the present study, an M20 peptidase T-like protein (PepTL) was hypothesized to be involved in Vsm maturation in *V. splendidus* JZ6 since mRNA encoding this peptidase was up-regulated under low-temperature conditions. The recombinant protein of PepTL was obtained and its proteolytic activity on Vsm was demonstrated *in vitro*. A Δ*pepTL* mutant was constructed to elucidate the function of PepTL in toxicity regulation of Vsm at different temperatures.

## Materials and methods

### Bacterial strains and growth conditions

The Yesso scallop (*Patinopecten yessoensis*) pathogen, *V. splendidus* strain JZ6 (Liu et al., 2013), was cultivated on Zobell 2216E agar at 20°C for 24 h. *E*. *coli* strains DH5α, BL21 (DE3), SY327 and S17-1 (Table 1) were cultured on LB agar at 37°C for 24 h.

**Table 1:** **Bacterial strains and plasmids used in the present study**.

**Strans/plasmids**	**Description^a^**	**References**
***VIBRIO SPLENDIDUS* STRAINS**		
JZ6	*V. splendidus* JZ6, a pathogenic agent of scallop	Liu et al., 2013
Δ*pepTL*	*pepTL* gene deletion mutant of *V. splendidus* JZ6	This study
***ESCHERICHIA COLI* STRAINS**		
DH5α	F^−^, φ80d*lac*ZΔM15, Δ(*lacZYA-argF*) U169, *deoR, recA*1, *endA*1, *hsdR*17 (${\text{r}}_{K}^{-}$, ${\text{m}}_{K}^{+}$), *phoA, supE44*, λ^−^, *thi*-1, *gyrA*96, *relA*1	Tiangen
BL21 (DE3)	F^−^, *ompT, hsdSB* (${\text{r}}_{B}^{-}$, ${\text{m}}_{B}^{-}$), *gal, dcm* (DE3)	Tiangen
SY327	Δ(*lac pro*), *argE*(Am), *recA56, rpoB*, λ *pir*	Ma et al., 2009
S17-1	Tp^r^, Sm^r^, *recA, thi, pro* (${\text{r}}_{K}^{-}$, ${\text{m}}_{K}^{-}$), *RP4:2-Tc:* MuKm, Tn7, λ *pir*	Ma et al., 2009
**PLASMIDS**		
pMD19-T simple	High copy number cloning vector, Amp^r^	TaKaRa
pET28a (+)	Prokaryotic expression vector, Kan^r^	Novagen
pK18mobsacB-Ery	Widely used gene knockout vector, Kan^r^, Ery^r^	Wang et al., 2015
pK18Ery-*pepTL*	pK18mobsacB-Ery containing the homologous fragment of *pepTL* gene of JZ6, Kan^r^, Ery^r^	This study

a *Amp^r^, ampicillin resistance; Ery^r^, erythromycin resistance; Kan^r^, kanamycin resistance*.

### Analysis of extracellular products (ECPs) from *V. splendidus* JZ6 at different temperatures

ECPs of *V*. *splendidus* JZ6 wild-type and mutant strains were obtained by using the cellophane method (Balebona et al., 1998). Strains were grown in Tryptic Soy Broth (TSB) medium supplemented with 2% w/v sodium chloride (NaCl) at 20°C for 12 h, and 200 μL of the cultures was spread onto Zobell 2216E agar plates, overlayed with sterile cellophane sheets and plates were incubated at 4, 10, 16, 20, 24, and 28°C for 24 h. Bacterial cells were harvested with sterile saline, and the cell suspensions were centrifuged at 12,000g, at 4°C for 10 min. The bacterial cells were collected and stored at −80°C prior to RNA extraction. The supernatants were filtered through 0.22 μm membrane filters and used as the crude ECPs (designated as P_4_, P_10_, P_16_, P_20_, P_24_, and P_28_). The total protein contents of the ECPs were measured following the bicinchonininc acid (BCA) method (Smith et al., 1985).

The concentration of the six ECPs samples were adjusted at 20 μg/μL, and 10 μL were loaded onto a 12% SDS-polyacrylamide gel for SDS-PAGE. Following electrophoresis, protein bands were visualized with Coomassie bright blue R250. Vsm in ECPs samples was detected by immunoblotting using a monoclonal antibody of JZ6 Vsm (Liu et al., 2016). The ECPs samples were transferred onto a sheet of nitrocellulose transfer membrane (Millipore, USA). The membrane was blocked with 5% skim milk powder solution overnight and incubated with anti-Vsm solution (1:1000, v/v) at room temperature (RT) for 1 h. After being washing with TBST (10 mmol/L Tris-HCl, pH 8.0, 100 mmol/L NaCl and 0.05% (w/v) Tween 20), the membrane was incubated with 1:2000 (v/v) horseradish peroxidase-conjugated anti-mouse IgG (Life Technologies, USA) at RT for 1 h. Finally, the membrane was incubated with SuperSignal®West Pico (Thermo Scientific, USA) and exposed to film. Mouses pre-immune serum was used as negative control.

### Mass spectrometry analysis of proteins in ECPs

After SDS-PAGE, protein bands from the ECPs of strain JZ6 were excised and sent to BGI (BGI technology service co., LTD, China) for mass spectrometry (MS) analysis. Proteins were digested with 0.01 μg/μL trypsin, and the resulting peptides were subjected to nanoelectrospray ionization followed by tandem mass spectrometry (MS/MS) in a LTQ Orbitrap Velos (Thermo, USA) coupled online to the HPLC. Raw data files acquired from the Orbitrap were converted into MGF files using Proteome Discoverer 1.2 (Thermo, USA), and protein identification was performed by using Mascot search engine 2.3.02 (Matrix Science, United Kingdom).

### RNA extraction and quantitative real-time PCR analysis

Total RNA was extracted with TRIzol reagent (Invitrogen, USA). Based on Promega M-MLV RT Usage information, the first-strand cDNA synthesis was carried out with the DNase I (Promega, USA)-treated total RNA and random primers (TaKaRa, Japan). Synthesis was performed at 42°C for 1 h, then terminated by heating at 95°C for 5 min.

The mRNA levels of the *vsm* and *pepTL* at different temperatures were validated by quantitative real-time PCR (qRT-PCR). Specific primers (P1/P2 for *vsm* and P3/P4 for *pepTL*) were designed according to the corresponding sequences in the genome of strain JZ6 (Table 2). The comparative C_T_ method (2^−ΔΔCT^ method) was used to analyze the expression level (Livak and Schmittgen, 2001). Two 16S rDNA primers for *V*. *splendidus*, P5 and P6 (Table 2), were used as internal control to verify successful transcription and to calibrate the cDNA template for corresponding samples. qRT-PCR was performed using Applied Biosystems 7500 (Life technologies, USA), and the collected data were analyzed with 7500 System SDS Software. The assay was conducted in a volume of 20 μL consisting 10 μL of 2 × SYBR® Premix Ex Taq™ II (TaKaRa, Japan), 0.8 μL of each forward and reverse primer (10 μmol/L), 0.4 μL of 50 × ROX reference dye, 2 μL of DNA extract (10 ng/μL) and 6 μL of nuclease-free water. The reaction was performed at 95°C for 30 s, 40 cycles of primer annealing at 95°C for 5 s, primer extension at 60°C for 31 s. Dissociation curve analysis of amplification products was performed at the end of each PCR to confirm that only one PCR product was amplified and detected. All data were given in terms of relative mRNA expressed as mean ± S.E. (*N* = 4).

**Table 2:** **The primers used in the present study**.

**Primer name**	**Sequence (5′-3′)**
**Vsm qRT-PCR**	
P1	AAGTCGCCCAAGTGGTGTATCT
P2	CGATGGGAAAGCTAGGGAAGT
**PepTL qRT-PCR**	
P3	TTCACAAACTGGCTGTCCCTAC
P4	GCCGATACCTGGCGTTACTG
**16S qRT-PCR**	
P5	TCGTGTYGTGARATGTTGGGT
P6	CCACCTTCCTCCRGTTTRTCA
**Vsm EXPRESSION**	
P7	GAATTCATGGCAGAAATGGTCAGAGTCG
P8	GAATTCATGCCAAAGCTGAATCGAGAACAAGC
P9	GAATTCATGGGACTAAACCACGCTAAAGCATTAG
P10	CTCGAGGACACCACAACTCGCATTAACG
**PepTL EXPRESSION**	
P11	CCATGGATATGGAACAACGTCTTGTAGAACATTTC
P12	GAATTCATGGAACAACGTCTTGTAGAACATTTC
**PepTL KNOCKOUT**	
P13	CTAGTCTAGAGAACAACGTCTTGTAGAACATTTCT
P14	GATGACCACCCCCGAGCGTAGATGTTGAAACCGT
P15	GGGGTGGTCATCTCACCCGCACATTCTTTCTATC
P16	ACGCGTCGACCGTCACGTAAGCCACAACAAA

### Expression and purification of recombinant proteins

Genomic DNA of *V*. *splendidus* JZ6 was extracted from 5 mL of an overnight culture using the DNeasy Blood and Tissue Kit according to the manufacturer's protocol (Qiagen, Germany). Specific primers P7, P8, and P9 (Table 2) were designed from the nucleotide sequences encoding the upstream fragment of Vsm's fungalysin/thermolysin propeptide (FTP) domain, protease inhibitory function (PepSY) domain and peptidase M4 domain, respectively. An *Eco*RI site sequence was added at 5′ end. P10 (Table 2) was designed as a reverse primer with an *Xho*I site sequence. PCR products were digested with *Eco*RI and *Xho*I (NEB, USA), and ligated into the same restriction enzymes sites of expression vector pET28a (+) (Merck, Germany).

The complete open read frame (ORF) encoding PepTL was amplified with primers P11 and P12 (Table 2) containing *Nco*I and *Eco*RI recongnition sequences at their 5′ end, respectively. The PCR product was digested with *Nco*I and *Eco*RI (NEB, USA), and ligated into the same restriction enzymes sites of expression vector pET22b (+) (Merck, Germany).

The recombinant plasmids were transformed into competent cell *E*. *coli* BL21 DE3 (Tiangen, China), and transformants were incubated in LB medium at 37°C with shaking at 220 rpm for 4 h. IPTG was added to the cell cultures to a final concentration of 1 mmol/L once the cultures reach at an OD_600_ of 0.4–0.6, and incubated at 18°C with shaking at 150 rpm for 24 h. The cultures were sonicated (200 W for 30 min) and centrifuged (12,000g, 4°C for 10 min) to obtain the supernatant containing soluble target proteins. Recombinant proteins (rVsmP1, rVsmP2, rVsmP3, and rPepTL) were purified with a Ni Sepharose column (Roche, Switzerland), and dialyzed against Tris buffer (20 mmol/L Tris-HCl, 150 mmol/L NaCl, pH 8.0) for 24 h. The resultant proteins were separated by SDS-PAGE, and visualized with Coomassie Bright Blue R250. The concentrations of purified soluble proteins were quantified by BCA method (Smith et al., 1985).

### Structure analysis and interaction prediction of protein PepTL and Vsm

The protein domains of PepTL and Vsm were predicted by the simple modular architecture research tool (SMART) (http://smart.embl-heidelberg.de/). The presumed structures of both PepTL and Vsm were modeled by using the prediction algorithm I-TASSER (http://zhanglab.ccmb.med.umich.edu/) and displayed by Jmol Viewer (version 14.4.4). The interaction between PepTL and Vsm was *in silico* performed and displayed by AutoDock Tools version 1.5.6.

### *In vitro* cleavage of Vsm in ECPs with rPepTL

The *in vitro* assay was carried out to assess the proteolytic activity of rPepTL. Three concentrations of rPepTL (5, 10 and 20 μg/μL) were screened in preliminary experiment, and 10 μg/μL of protein was determined to be an optimal concentration. For the experimental group, 50 μL of rPepTL solution containing 10 mmol/L ZnCl_2_ was mixed with 50 μL of ECPs P_28_ (20 μg/μL) at 20°C for 1 h. Fifty microliter of ECPs P_28_ and ECPs P_10_ was used as negative and positive control, respectively. After the reaction, the mixtures were separated by SDS-PAGE and analyzed for reaction products as described in Section Analysis of Extracellular Products (ECPs) From *V. splendidus* JZ6 at Different Temperatures.

### Construction of a *pepTL* gene deletion mutant of *V. splendidus* JZ6

The suicide plasmid, pK18mobsacB-Ery (Wang et al., 2015), was used for deletion of the region encoding the active site in PepTL. Two pairs of primers (P13/P14 and P15/P16) were used to amplify the upstream and downstream DNA sequences of the target region from strain JZ6 genomic DNA. The PCR fragments of 170 and 220 bp were purified and fused in an overlap PCR reaction using primers P13 and P16 (Table 2). The fused segment was sequenced and digested with *Xba*I/*Sal*I (NEB, USA), cloned into the same sites of pK18mobsacB-Ery, and then transformed into *E*. *coli* stains SY327 and S17-1. Suicide plasmid pK18Ery-*pepTL* was mobilized from *E*. *coli* S17-1 into *V*. *splendidus* JZ6 by intergeneric conjugation.

After mating, cells were spread on 2216E plates containing erythromycin (25 μg/mL) to select for clones in which the suicide vector pK18Ery-*pepTL* had been integrated into the JZ6 genome via a single crossover event. The mutants were then grown at 20°C with shaking in 2216E medium without any antibiotics for 8 h. To select mutants in which a second recombination event had occurred, the culture was diluted and spread on 2216E medium containing 10% sucrose and incubated at 20°C for 24–36 h. Single colonies were replica-plated onto 2216E and erythromycin containing 2216E plates, and colonies sensitive to erythromycin (25 μg/mL) were collected and confirmed by PCR followed by DNA sequencing.

### Statistical analysis

The significant differences among groups were subjected to one-way analysis of variance (one-way ANOVA) and multiple comparisons by using SPSS 16.0 program. Statistically significant difference was designated at *p* < 0.05 or *p* < 0.01.

## Results

### Vsm in ECPs from strain JZ6 is present in two forms

**Figure 1:**
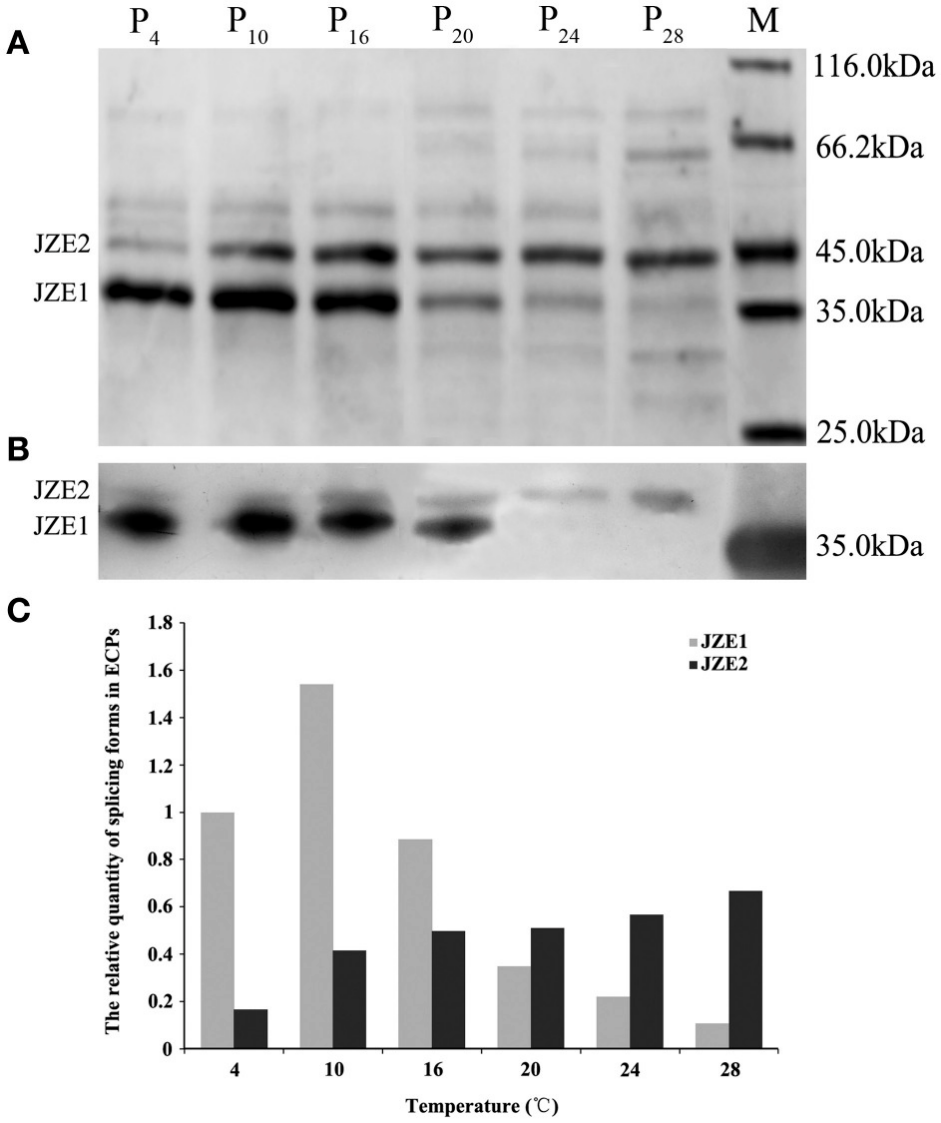
**Extracellular products (ECPs) of *V*. *splendidus* JZ6 cultured at 4, 10, 16, 20, 24, and 28°C, respectively, analyzed by SDS-PAGE and Western-blotting**. **(A)** P_4_, ECPs of JZ6 cultured at 4°C. P_10_, ECPs of JZ6 cultured at 10°C. P_16_, ECPs of JZ6 cultured at 16°C. P_20_, ECPs of JZ6 cultured at 20°C. P_24_, ECPs of JZ6 cultured at 24°C. P_28_, ECPs of JZ6 cultured at 28°C. Lane M, protein molecular weight standards. **(B)** Western-blotting detected the extracellular metalloprotease (Vsm) of *V*. *splendidus* with monoclonal antibody of JZ6 Vsm diluted 1:1000. **(C)** The relative quantities of two bands assessed with Image Lab Software.

In our previous study, we demonstrated that Vsm from strain JZ6 was presented in the extracellular protein fraction in two forms differing in their molecular weight (Liu et al., 2016). In the present study, the differences in ECPs from strain JZ6 cultured at different temperatures (4, 10, 16, 20, 24, and 28°C) were monitored by SDS-PAGE. Two bands (JZE1:~35kDa and JZE2: ~45kDa) were observed in these ECPs, and their relative quantities were assessed with Image Lab Software (Bio-Rad, USA). At 4°C, JZE1 was the dominant band observed in ECPs P_4_, with an intensity 5.88-fold that of JZE2 (~45kDa) (Figures 1A,C). As the temperature increased, the relative quantity of JZE1 increased at 10°C (1.54-fold of JZE1 in ECPs P_4_) and then decreased from 0.89- to 0.11-fold of the level of JZE1 in ECPs P_4_ as the temperature increased from 16 to 28°C (Figures 1A,C). The concentration of JZE2 in ECPs increased 0.42-, 0.49-, 0.51-, 0.57-, and 0.67-fold that of JZE1 in ECPs P_4_ at 10, 16, 20, 24, and 28°C, respectively (Figures 1A,C). Western-blotting with a monoclonal antibody against JZ6 Vsm also revealed two main bands in ECPs of strain JZ6 (Figure 1B), and their intensity changes were consistent with the tendency observed in the SDS-PAGE assay.

Two bands of JZE1 and JZE2 were analyzed by LC-ESI-MS/MS mass spectrometry. Eight and eleven specific peptide fragments detected in JZE1 and JZE2, respectively, and all of them were identified as Vsm from strain JZ6. The eight specific peptide fragments from JZE1 were located in the M4 domain, and so were eight of the eleven fragments from JZE2. The other three peptides from JZE2 were assigned to the PepSY domain (Table 3). Thus, JZE1 was generated from JZE2 by cleavage.

**Table 3:** **The peptide fragments identified by mass spectrum analysis**.

**Source**	**Peptide fragment**	**Protein**	**Location^a^**	**Domain^b^**
**JZE1**				
	AAADMGYVVADVEDAFNTVGVNASCGVTPPTGNVLTK	Vsm	477–513	M4
	GNVDWVVGSDIFK	Vsm	382–394	M4
	KGFEIFTVANQLYWTANSTFDAGACGVAK	Vsm	448–476	M4
	SEGGLRYFDQPSK	Vsm	395–407	M4
	SIDHASQYYDGLNVHLSSGVYNR	Vsm	411–433	M4
	YEYGSDFPSFPIDK	Vsm	212–225	M4
	YFDQPSK	Vsm	401–407	M4
	YINGAYSPLNDAHYFGNVVFDMYK	Vsm	267–290	M4
**JZE2**				
	AAADMGYVVADVEDAFNTVGVNASCGVTPPTGNVLTK	Vsm	477–513	M4
	DGRSIDHASQYYDGLNVHLSSGVYNR	Vsm	408–433	M4
	EWMNTSPLTFQLTMR	Vsm	291–305	M4
	GKKSIENKNAKLMVRLDENQTA	Vsm	136–157	PepSY
	GNVDWVVGSDIFKSEGGLR	Vsm	382–400	M4
	SIDHASQYYDGLNVHLSSGVYNR	Vsm	411–433	M4
	TTRYEYGSDFPSFPIDK	Vsm	209–225	M4
	YFDQPSK	Vsm	401–407	M4
	YFIDATTGDVLQKWNGLNHAK	Vsm	177–197	PepSY
	YINGAYSPLNDAHYFGNVVFDMYK	Vsm	267–290	M4
	YLVDFFIASSMPERPF	Vsm	161–176	PepSY

a *The location of peptide fragments in Vsm protein. The full-length of Vsm protein was 607 amino acids*.

b *The identified peptide fragments located in domains of Vsm*.

### The *Vsm* and *pepTL* mRNA levels decrease with increasing temperatures

**Figure 2:**
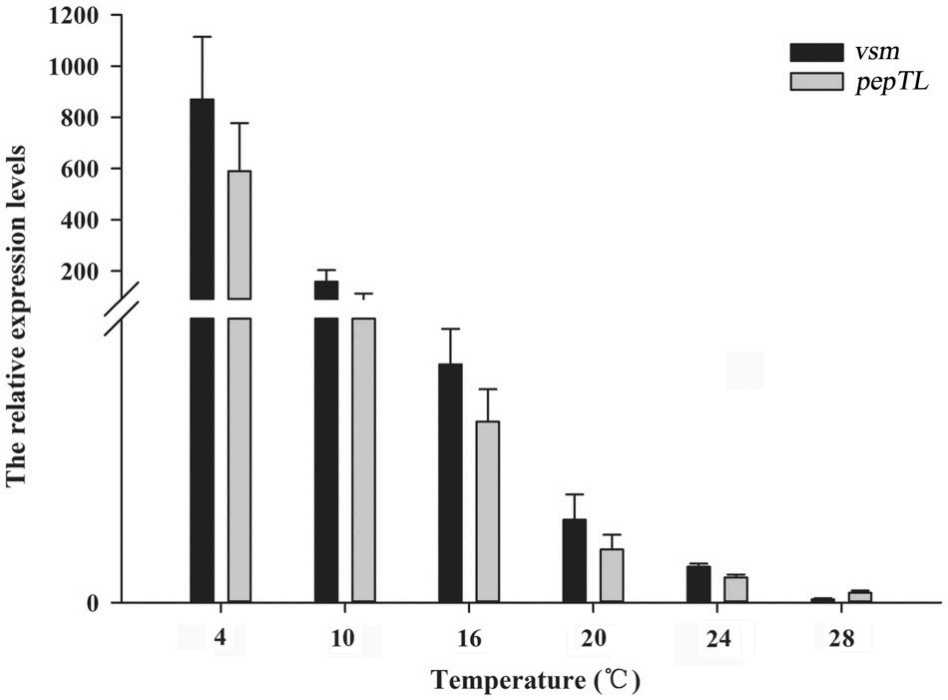
**Relative *vsm* and *pepTL* mRNA expression levels in strain JZ6 at 4, 10, 16, 20, 24, and 28°C as determined by qRT-PCR**.

The relative expression levels of *vsm* mRNA at 4, 10, 16, 20, 24°C were significantly higher (*p* < 0.01) than that at 28°C, with the highest level at 4°C (836.82-fold of that at 28°C), and then gradually decreased with the temperature increasing from 10 to 24°C (Figure 2). Similarly, the relative expression level of *pepTL* was highest at 4°C (191.38-fold, *p* < 0.01), and gradually decreased from 29.25-fold (*p* < 0.01) at 10°C to 2.59-fold (*p* < 0.05) at 24°C, finally reaching its lowest level at 28°C (Figure 2).

### Modeling the structure of PepTL and Vsm

**Figure 3:**
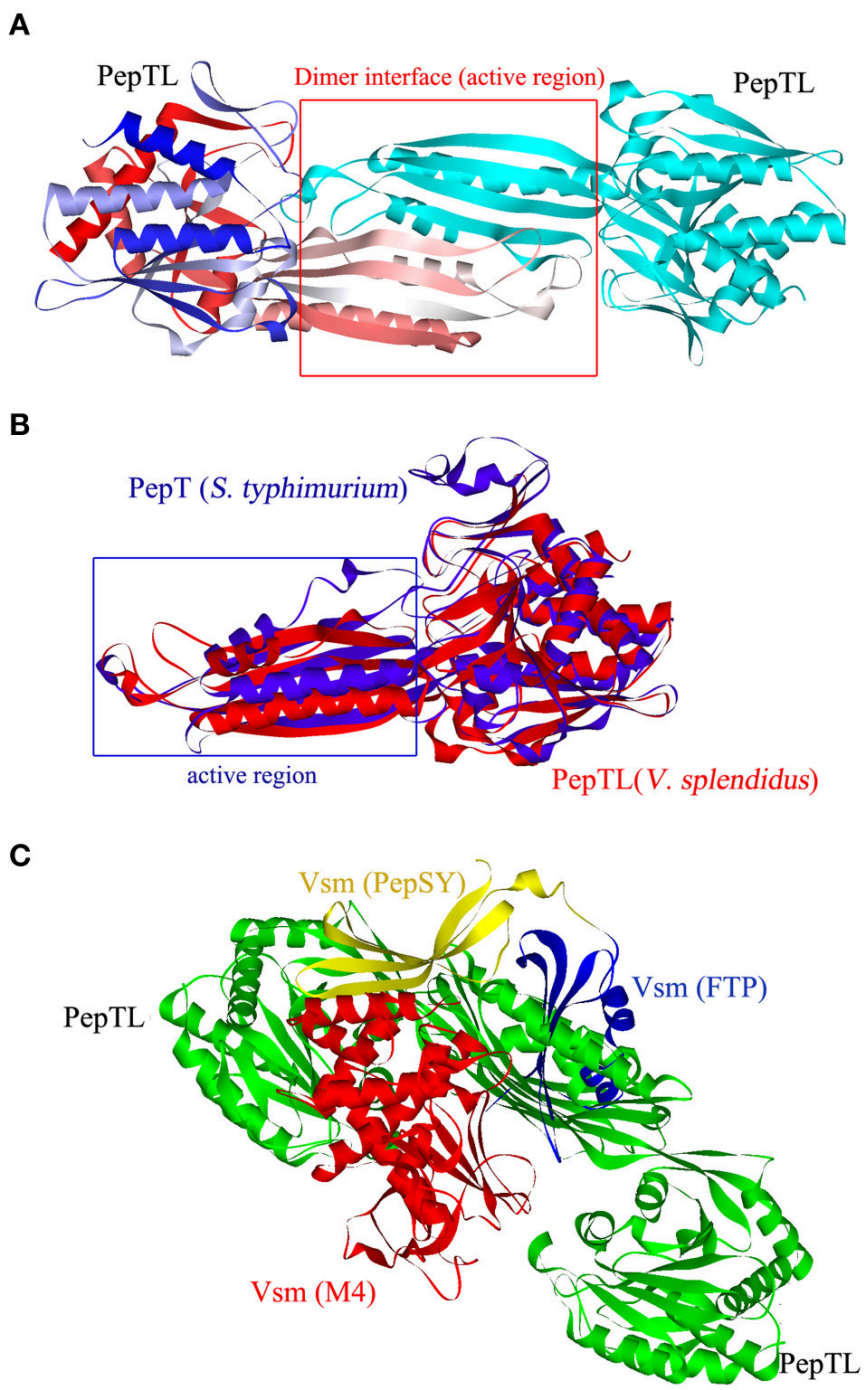
**Three dimensional structures of PepTL and Vsm protein. (A)** Ribbon diagram of the PepTL homodimer. **(B)** The superposition view of *S. typhimurium* PepT (blue) and *V*. *splendidus* JZ6 PepTL (red) structures. **(C)** The interaction of PepTL and Vsm displayed by AutoDock Tools.

PepTL is predicted to perform its proteolytic activity as a homodimer (Figure 3A). A structure similar to that of *S. typhimurium* peptidase T (PepT, PDB No.1fno), was predicted with 10 α-helix and 15 β-sheet elements, but the conformation of dimerization domain from Ala176 to Phe291 in PepTL was significantly different from that of PepT (Glu206-Asn321) (Figure 3B). The 3D structure of Vsm consisted of the FTP domain, PepSY domain and two M4 domains (Figure 3C). *In silico* analysis of interaction by AutoDock Tools revealed that the homodimer of PepTL could tightly bind to Vsm, and the binding sites were located in the gap between PepSY and M4 domains of Vsm (Figure 3C).

### *In vitro* proteolytic activity of PepTL

**Figure 4:**
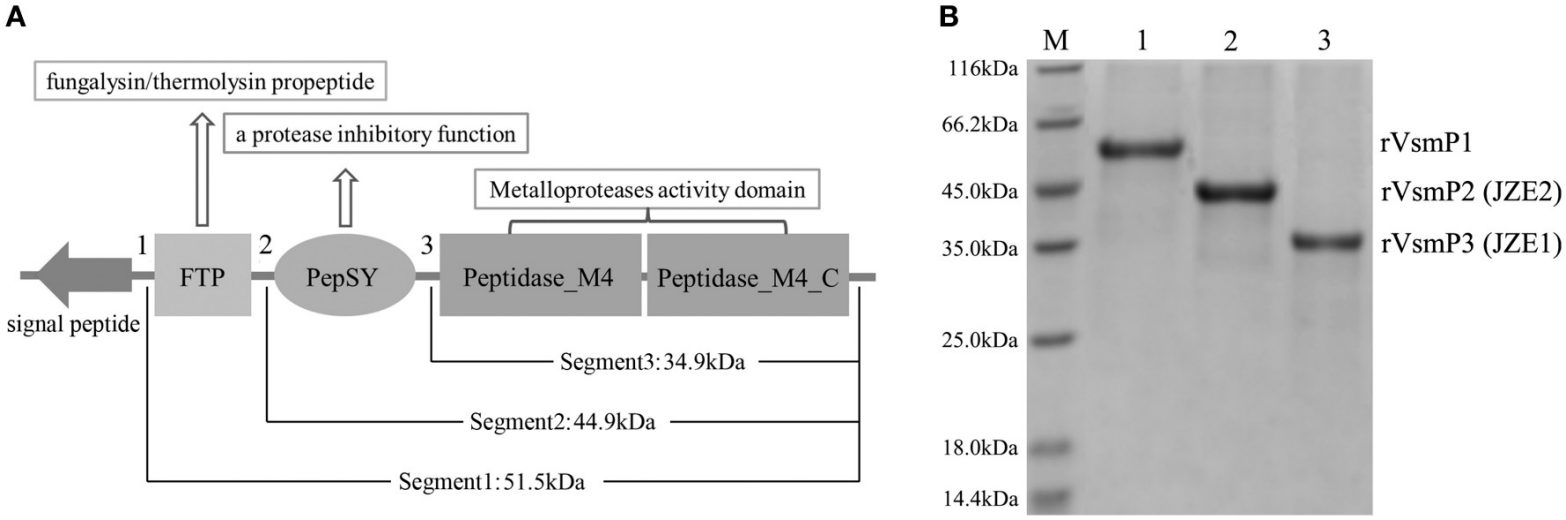
**Structure characteristics of the different protein segments of Vsm in *V*. *splendidus* JZ6. (A)** Scheme showing protein domains present in full length of Vsm. **(B)** purified recombinant proteins of the different Vsm forms analyzed by SDS-PAGE. Line M, protein molecular standard. Line 1, rVsmP1. Line 2, rVsmP2. Line 3, rVsmP3.

Three protein segments of Vsm containing different functional domains were expressed to confirm the composition and molecular weight of JZE1 and JZE2. Segment 1 of Vsm (rVsmP1) consisted of 478 amino acids, containing FTP, PepSY and two Peptidase_M4 domains with a predicted molecular weight of ~51.50 kDa (Figure 4A). Segment 2 (rVsmP2) was composed of PepSY and two Peptidase_M4 domains (389 amino acids with a predicted molecular weight of ~44.93 kDa). Segment 3 (rVsmP3) only had two Peptidase_M4 domains (312 amino acids), and its predicted molecular weight was ~34.90 kDa. These recombinant proteins were expressed in *E*. *coli* BL21 (DE3) and purified by Ni Sepharose column. After SDS-PAGE analysis, the molecular weights of purified proteins were consistent with their theoretical values (Figure 4B), indicating that rVsmP3 shared a similar protein size with JZE1, and rVsmP2 had the same domain architectures as JZE2.

**Figure 5:**
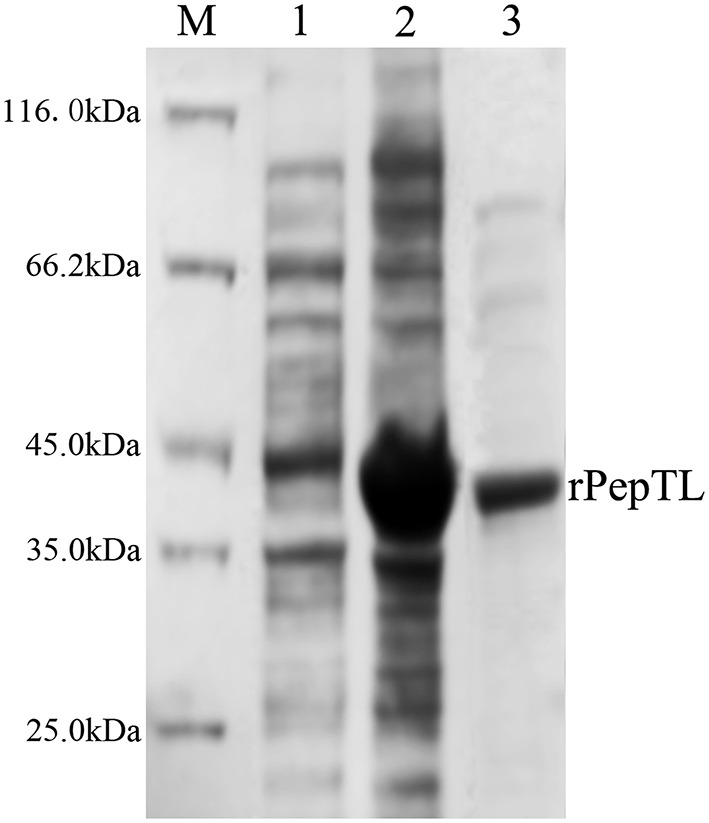
**SDS-PAGE analysis of the recombinant PepTL protein (rPepTL)**. Lane M, protein molecular standard. Lane 1, uninduced rPepTL (the whole cell lysate). Lane 2, induced rPepTL. Lane 3, purified rPepTL.

**Figure 6:**
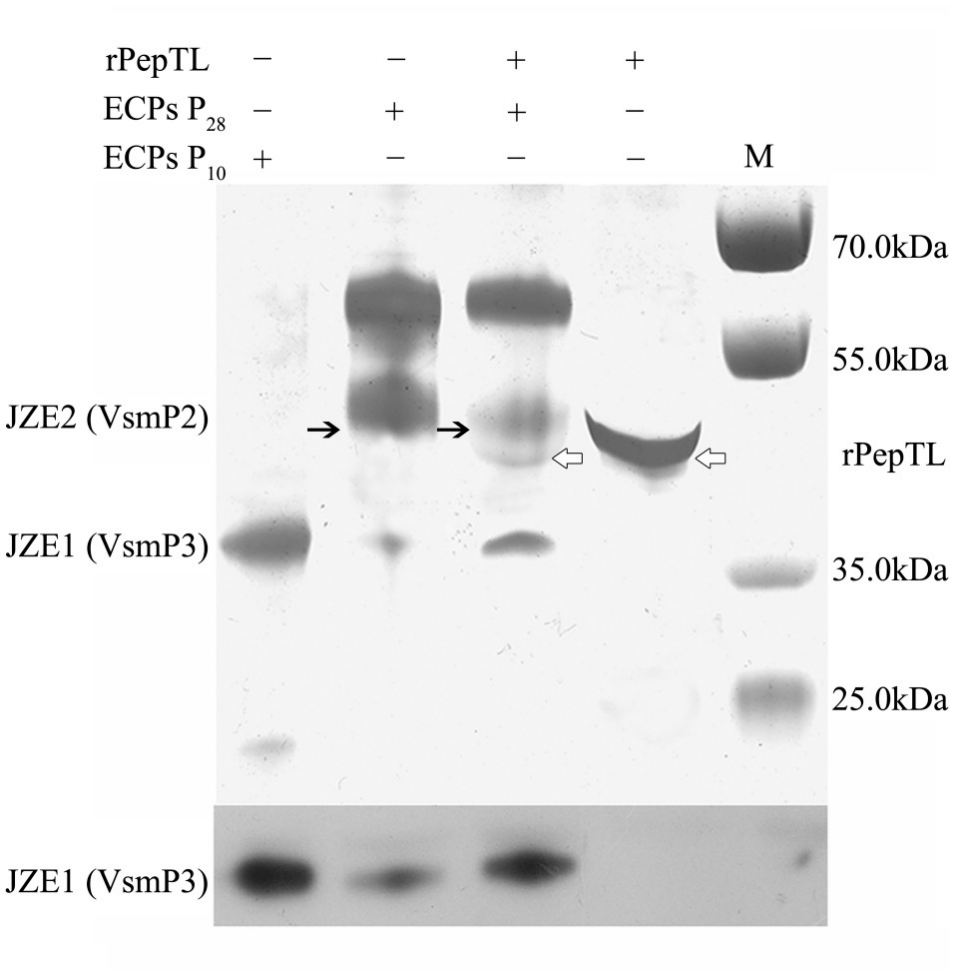
**rPepTL-induced cleavage of Vsm in ECPs as determined by SDS-PAGE**. Line 1, sample with only ECPs of strain JZ6 cultured at 10°C. Line 2, sample with only ECPs of strain JZ6 cultured at 28°C. Line 3, sample with both rPepTL and ECPs of strain JZ6 cultured at 28°C. Line 4, sample with only rPepTL. Line M, protein molecular weight standards.

The full-length ORF of *pepTL* was of 1107 bp, encoding a polypeptide of 368 amino acids with a predicted molecular weight of ~39.30 kDa. Four different domain architectures of PepTL were analyzed by SMART, and the Peptidase_M20 domain was considered as the PepTL with *E*-value 3.8 × 10^−13^. Recombinant PepTL was expressed in *E*. *coli* BL21 (DE3) utilizing pET22b (+). After 24 h IPTG induction, the whole cell lysate was analyzed by SDS-PAGE and a distinct band was revealed with a molecular weight of ~40 kDa (Figure 5, Lane 2) which was in agreement with the predicted molecular mass. The purified and refolded rPepTL was of the same molecular weight (Figure 5, Lane 3). After incubation of ECPs P_28_ with rPepTL at 20°C for 1 h, the intensity of the band corresponding to JZE2 dramatically decreased and the intensity of the JZE1 band increased (Figure 6). Furthermore, the quantity of rPepTL was less than that in the rPepTL only group (Figure 6). The changes in the intensity of the bands corresponding to JZE1 were also observed in immunoblotting with the monoclonal antibody of Vsm (Figure 6). No autocatalytic activity of ECPs P_10_ and P_28_ was observed.

### Participation of PepTL in activation of Vsm *in vivo*

**Figure 7:**
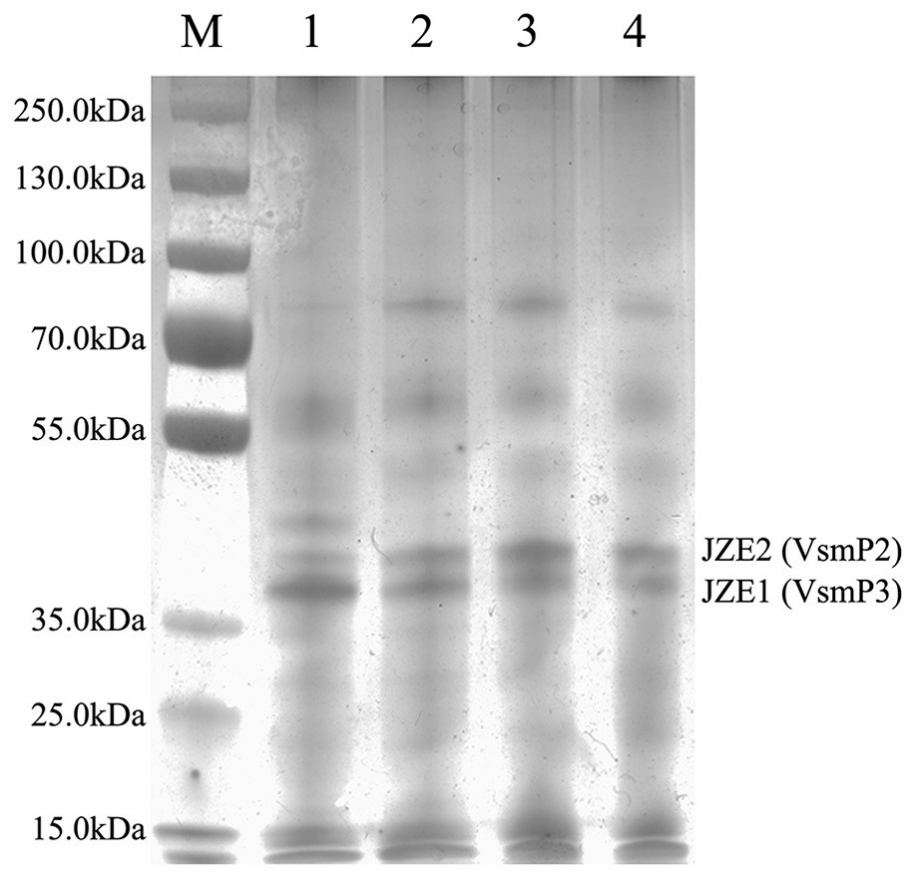
**ECPs of *V*. *splendidus* JZ6 and Δ*pepTL* mutant cultured at 10 and 28°C, respectively, analyzed by SDS-PAGE**. Lane M, protein molecular weight standards. Line 1, ECPs of JZ6 cultured at 10°C. Line 2, ECPs of JZ6 cultured at 28°C. Line 3, ECPs of Δ*pepTL* mutant cultured at 10°C. Line 4, ECPs of Δ*pepTL* mutant cultured at 28°C.

The function of PepTL was also tested by deleting the *pepTL* gene from strain JZ6. ECPs from both wide type and Δ*pepTL* mutant were collected and subjected to SDS-PAGE post-culture at 10 and 28°C. Compared with the wild type (Figure 7, Line 1 and 2), the bands for Vsm in ECPs were not significantly different when the Δ*pepTL* mutant was cultured at 10 and 28°C (Figure 7, Line 3 and 4). The banding patterns of JZE1 and JZE2 were completely consistent with ECPs P_28_ of wild type, indicating that *pepTL* gene was crucial for the transformation from JZE2 to JZE1.

## Discussion

*V*. *splendidus* JZ6 was isolated from a diseased Yesso scallop (*P*. *yessoensis*) during winter and shown to cause higher mortality of scallops at 10°C than at 28°C (Liu et al., 2013). Its unique biological properties, such as the regulation of its pathogenicity by temperature distinguish this strain from other *V*. *splendidus* strains and *Vibrio* spp. (Liu et al., 2013, 2016). Our previous studies indicated that the virulence of strain JZ6 was mainly determined by the quantity and toxicity of Vsm, which was dependent on the temperature (Liu et al., 2016). Although comparative transcriptome analysis was able to demonstrate temperature regulation of Vsm expression, it did not reveal the mechanism of its post-translational modifications.

Vsm of *V*. *splendidus* strain JZ6 shared high sequence and structural similarities with thermolysin-like metalloproteases (TLPs) in M4 family, such as vibriolysin of pathogenic *Vibrio* spp. (Peters et al., 1982; Miyoshi et al., 1987; Lutfullah et al., 2008; Iqbal et al., 2011). Extracellular metalloproteases are usually synthesized as inactive zymogens with the catalytic domain inhibited by a propeptide. Removal of the propeptides is dependent on other proteases or an autocatalytic process (Nickerson et al., 2008; Gao et al., 2010). Unlike most of TLPs, a typical autocatalytic process was not observed during the maturation of Vsm, and another peptidase was predicated to involve in this process of Vsm. In the present study, two forms (JZE1 and JZE2) of Vsm were identified in ECPs of strain JZ6. JZE1 only included the activity region (peptidase_M4 domain) of Vsm with a molecule weight of ~35kDa. JZE2 was containing the activity region of Vsm and the PepSY domain (~45kDa). JZE1 was expressed at higher levels in ECPs at low temperatures (highest at 10°C), and its concentrations gradually decreased with an increase in temperatures. On the contrary, JZE2 was detected with the highest concentration at 28°C and lowest at 4°C. As a low-temperature pathogenic bacterium, the pathogenicity of strain JZ6 and the toxicity of Vsm at 10°C were higher than those at 28°C (Liu et al., 2013, 2016). These observations indicated that JZ1 containing only M4 domain was likely the key modular part of Vsm with toxicity.

PepTL was annotated as a peptidase T-like protein of *V. splendidus* by BlastP analysis. It displayed a parallel expression pattern with Vsm at 10 and 28°C in strain JZ6 (Liu et al., 2016). PepTL containes similar secondary structure elements as PepT of *S*. *typhimurium* (Håkansson and Miller, 2002), thus it possesses the structural base of peptidase T proteins although the tertiary structures of PepTL and PepT are different from each other in their dimerization domain according to molecular overlay analysis. The conformation of PepTL from Ala176 to Phe291 was predicted to deviate significantly from the corresponding motif (Glu206-Asn321) of PepT. This observation suggests a different function of PepTL compared with PepT (Lindner et al., 2003). The 3D structures of PepTL and Vsm were predicted with homology modeling, and the interaction between PepTL and Vsm was analyzed *in silico* by AutoDock Tools. Although the dimerization domain of PepTL was different from that of PepT, the homodimer was still predicted to exist in PepTL. After the analog calculation, the dimer interface of PepTL was found to closely bind to the Vsm and to fit into the space between PepSY and M4 domains. This result is accordant with the observation of most M20 peptidases which commonly form the homodimers to bind polypeptides in some proteins, and whose active region is situated mainly at the interface between two protein molecules (Lindner et al., 2003). Therefore, PepTL possesses the structural foundation for binding and catalyzing the Vsm cleavage.

In order to determine the proteolytic activity of PepTL, Vsm degradation was analyzed via *in vitro* and *in vivo* experiments. In the *in vitro* reaction, rPepTL degraded JZE2 in ECPs P_28_, and the concentration of JZE1 increased considerable compared to the negative control. The function of PepTL was further verified with the Δ*pepTL* mutant *in vivo*. Compared with the wild type of strain JZ6, there was no difference in the bands corresponding to Vsm forms in ECPs of the Δ*pepTL* mutant at 10 and 28°C. These results demonstrate that PepTL of strain JZ6 indeed catalyzes the maturation of Vsm by removing the PepSY inhibitor domain from the M4 domain. Furthermore, since the deleted fragment of *pepTL* gene encodes the dimer interface, it is likely that homodimer formation is crucial for the peptidase activity of PepTL.

## Author contributions

RL is the first author of this manuscript, who is mainly responsible for the experimental design and most of the experimental results. LQ is the corresponding author, who is responsible for the guidance and the modification of this manuscript. QC is one of coauthors for this manuscript, who is responsible for the experiment of proteins. HZ is one of coauthors for this manuscript, who is responsible for the experiment of microbial genetic operations. LW is one of coauthors for this manuscript, who is responsible for the experiment of structural analysis. LS is the corresponding author, who is responsible for the guidance and the modification of this manuscript.

### Conflict of interest statement

The authors declare that the research was conducted in the absence of any commercial or financial relationships that could be construed as a potential conflict of interest.
